# Effects of an LPSO Phase Induced by Zn Addition on the High-Temperature Properties of Mg-9Gd-2Nd-(1.5Zn)-0.5Zr Alloy

**DOI:** 10.3390/ma17164075

**Published:** 2024-08-16

**Authors:** Ming Li, Mengling Yao, Liangzhi Liu, Xiaoxia Zhang, Zhihui Xing, Xiangsheng Xia, Peng Liu, Yuanyuan Wan, Qiang Chen, Hongxia Wang

**Affiliations:** 1 Southwest Technology and Engineering Research Institute, Chongqing 400039, China; liming9131217@163.com (M.L.); yaomengling59@163.com (M.Y.); 13650536549@163.com (Z.X.); liuphust@163.com (P.L.); 15215166054@163.com (Y.W.); 2009chengqiang@163.com (Q.C.); 2 Shanxi Key Laboratory of Advanced Magnesium Based Materials, School of Materials Science and Engineering, Taiyuan University of Technology, Taiyuan 030024, China; 13212009280@163.com (L.L.); 18834167996@163.com (X.Z.)

**Keywords:** rare earth magnesium alloy, LPSO phase, microstructure, high-temperature performance

## Abstract

In this study, we prepared Mg-9Gd-2Nd-0.5Zr, referred to as alloy I, and Mg-9Gd-2Nd-1.5Zn-0.5Zr, referred to as alloy II. The effects of a long-period stacking ordered (LPSO) phase induced by Zn addition on the high-temperature mechanical properties and fracture morphology of alloy I and alloy II at different temperatures (25 °C, 200 °C, 225 °C, and 250 °C) were studied using optical microscopy (OM), scanning electron microscopy (SEM), energy-dispersive spectroscopy (EDS), electron backscatter diffraction (EBSD), and transmission electron microscopy (TEM). The results indicate that Mg_5_RE at the crystal boundary of the as-cast alloy I transformed into (MgZn)_3_RE (as-cast alloy II) by the addition of Zn. After solid solution treatment, the secondary phase in alloy I completely disappeared, and there were still residual secondary phases in block-like and needle-like structures in alloy II, while layered LPSO phases precipitated in the matrix. During the high-temperature tensile test, the yield and tensile strength of alloy I decreased significantly with the increase in temperature, while the elongation increased. Compared to alloy I, the yield strength of alloy II with an LPSO phase showed an increasing trend at 25 °C~200 °C and then decreased when the temperature reached around 250 °C. The thermal stability was significantly enhanced, and the elongation was also higher than that of alloy I. As the temperature increased, the fracture surface of alloy I showed increased folding, bending of scratches, and crack enlargement. However, the fracture surface of alloy II remained largely unchanged, with only minor wrinkles and cracks appearing at temperatures reaching 250 °C.

## 1. Introduction

Magnesium alloys have the characteristics of low density, high specific strength, and good processability, which give them wide application prospects in automotive, aerospace, and electronic products and other fields [1,2]. However, traditional magnesium alloys have poor stability at high temperatures and cannot meet the requested material properties in complex environments, which poses certain obstacles to the widespread application of magnesium alloys as structural materials in related fields.

In recent years, studies have shown [3] that Mg-Gd-Nd rare earth magnesium alloys usually have high tensile and yield strength, especially aging alloys, which have been further strengthened. However, although rare earth (RE) magnesium alloys have high strength after heat treatment, their plasticity is relatively poor (generally <10%) [4,5]. In order to improve the plasticity of an alloy, the common method is to use plastic deformation to refine the particles, but for complex parts that are difficult to deform, the performance can only be improved through alloying and heat treatment [6]. Research has found that [7,8,9] the long-period stacking ordered (LPSO) phase itself has a certain degree of plasticity. When subjected to stress and deformation, it can coordinate intragranular dislocations by forming twisted bands, avoid stress concentration, prevent crack formation, and, to some extent, improve the plasticity of the material. By adding Zn to magnesium alloys in a certain proportion with RE elements, it is possible to induce the formation of a long-period stacking ordered (LPSO) phase in the subsequent heat treatment process [10]. According to previous research [11], with the increasing Zn content, the strength of the alloy progressively enhances. However, when the Zn content reaches 2%, the plasticity is relatively poor. Therefore, the Zn content is established at 1.5%. Meanwhile, Mg-Gd-Zn series alloys can precipitate a 14H-type LPSO phase after undergoing a subsequent heat treatment [12,13]. Research has found that the LPSO phase can significantly improve the high-temperature thermal stability and ductility of magnesium alloys, resulting in excellent strength and toughness. Therefore, the LPSO phase shows a great application prospect in improving the overall high-temperature properties of magnesium alloys.

This study takes Mg-9Gd-2Nd-0.5Zr as experimental alloy I and Mg-9Gd-2Nd-1.5Zn-0.5Zr as alloy II to study the microstructure changes in alloy I and alloy II after high-temperature fracture and the effects of an LPSO phase on the high-temperature performance of the alloys. It provides theoretical guidance for the future development of Mg-RE alloys with excellent high-temperature properties.

## 2. Experimental Materials and Methods

We used 99.9% (wt.%) pure Mg, 99.9% (wt.%) pure Zn, Mg-30Gd (wt.%) and Mg-30Nd (wt.%) intermediate alloys, and Mg-30Zr (wt.%) intermediate alloy as raw materials. Before melting, the crucible needed to be placed in a well-type resistance furnace and preheated to 300 °C. After preheating, the crucible was coated on the inner wall and then put back into the resistance furnace for drying. After finishing the preparation work, pure magnesium was added into the crucible. An SF_6_ and CO_2_ protective gas, the volume ratio of which was 1:99, was injected into crucible for the protection of pure magnesium from overheating. The temperature was raised to 720 °C and maintained for 30 min. After completely melting the pure magnesium, the temperature was raised to 780 °C, and the Mg-30Gd intermediate alloy was added into the crucible. After melting sufficiently, the temperature was reduced to 750 °C, and the Mg-30Nd and Mg-30Zr intermediate alloys were added and maintained for 30 min. The melt was poured into a mold and then cooled to 25 °C. Finally, alloy I was obtained. Under the above conditions, it was cooled to 730 °C, and pure Zn was added for another 30 min to complete the preparation of alloy II. The dimensions of the ingots for the two alloys were 140 mm × 100 mm × 20 mm.

The heat treatment process involved heating the sample in a box-type resistance furnace (SX2-10-13 type, Suzhou Chenguang Electric Furnace Manufacturing Co., Ltd., Suzhou, Jiangsu, China). Alloy I was first kept at 525 °C for 5 h for a solid solution treatment, and then quenched in water at a temperature of 25 °C. In order to obtain the LPSO phase, alloy II was first kept at 525 °C for 4 h, and then cooled down to 400 °C in the furnace. It remained for 4 h at 400 °C, followed by water quenching and cooling. Then, water-quenched alloy I and alloy II samples were subjected to an aging treatment. They were kept at 250 °C for 4 h and then air-cooled to room temperature.

**Figure 1:**
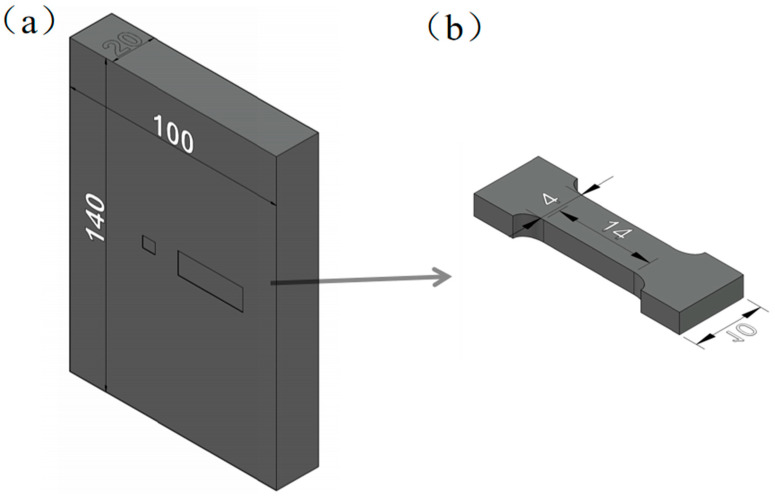
Sampling locations and size of high-temperature tensile sample: (**a**) size of ingot and sampling locations; (**b**) size of high-temperature tensile sample.

In order to ensure the representativeness and accuracy of the test, the observation sample was generally taken as close as possible to the central area of the casting. The sampling position is shown in Figure 1a. When observing the metallographic structure, the sample was polished using #800-2000 sandpaper in order from coarse to fine, respectively. We used #3000 sandpaper for water grinding in flowing water and then used a polishing machine to polish the sample and sprayed anhydrous ethanol to dry it. After drying, we used nitric acid reagent (96 mL of ethanol, 4 mL of nitric acid) to corrode the sample. The microstructure and phase structure of the alloy were characterized using a Leica DM 2700M optical microscope (Beijing Shunxian Hengye Technology Co., Ltd., Beijing, China. Tescan (China) Co., Ltd., Shanghai, China.) and TESCANMIRA3LMH scanning electron microscope (Beijing Shunxian Hengye Technology Co., Ltd., Beijing, China. Tescan (China) Co., Ltd., Shanghai, China.) equipped with an energy-dispersive spectrometer. The area fraction of the secondary phases was evaluated using Image-ProPlus 6.0 software (Image-Pro Plus 6.0.).

An MTS (E45.105) electronic material testing machine(MTS Industrial Systems (China) Co., Ltd., Shanghai, China) was used to perform high-temperature tensile tests on alloy I and alloy II after the aging treatment. According to research [14,15], the high-temperature service temperature of magnesium alloys is generally 50–200 °C higher than room temperature. Therefore, the temperatures for the tensile tests were set to 25 °C and around 225 °C. The parameters were set as 25 °C, 200 °C, 225 °C, and 250 °C, and the tensile strain rate was 5% (strain rate of 0.01 s^−1^). To ensure the reliability of the tensile experimental data, it was necessary to repeat the experiment 3 times and calculate the average value of the obtained data. The sampling locations and dimensions of the tensile specimens are illustrated in Figure 1a,b.

## 3. Results and Discussion

### 3.1. Microstructure of the Cast Alloys

**Figure 2:**
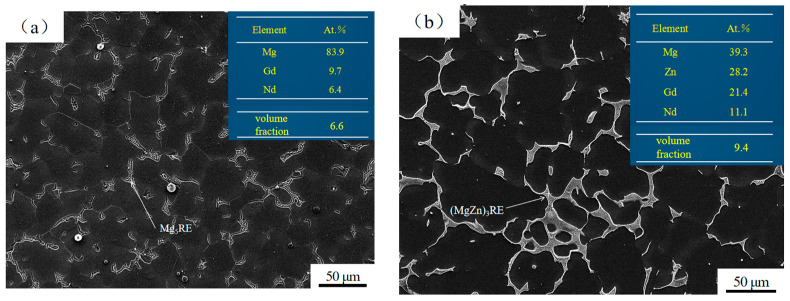
SEM pattern and phase composition of as-cast alloy structures: (**a**) alloy I; (**b**) alloy II.

Figure 2 shows a scanning electron microscopy (SEM) (Tescan (China) Co., Ltd., Shanghai, China.) diagram and phase composition of the as-cast alloys I and II. Figure 2a shows that alloy I consisted of an α-Mg matrix (the black area) and an irregular strip-shaped secondary phase (the white area) widely distributed along the grain boundaries. energy-dispersive spectroscopy (EDS) analysis of the white area revealed that it was primarily composed of Mg, Gd, and Nd, with an atomic (Gd+Nd)/Mg ratio close to 1:5, indicating that it was a Mg_5_RE(Gd,Nd) phase. The volume fraction statistics of the white area showed that it accounted for 6.6% of the total volume. As can be seen in Figure 2b, alloy II consisted of an α-Mg matrix, and the secondary phase was distributed along the grain boundary in a semi-continuous mesh-like shape. EDS analysis of the secondary phase showed that it was mainly composed of Mg, Zn, Gd, and Nd, with an atomic (Gd+Nd)/(Mg+Zn) ratio close to 1:3, indicating that it was a (Mg,Zn)_3_RE(Gd,Nd) phase. The volume fraction statistics of the secondary phase showed that it accounted for 9.4% of the total volume. Compared to alloy I, the addition of Zn significantly changed the morphology and quantity of the secondary phase in the alloy.

### 3.2. Microstructure of the Solid Solution Alloys

**Figure 3:**
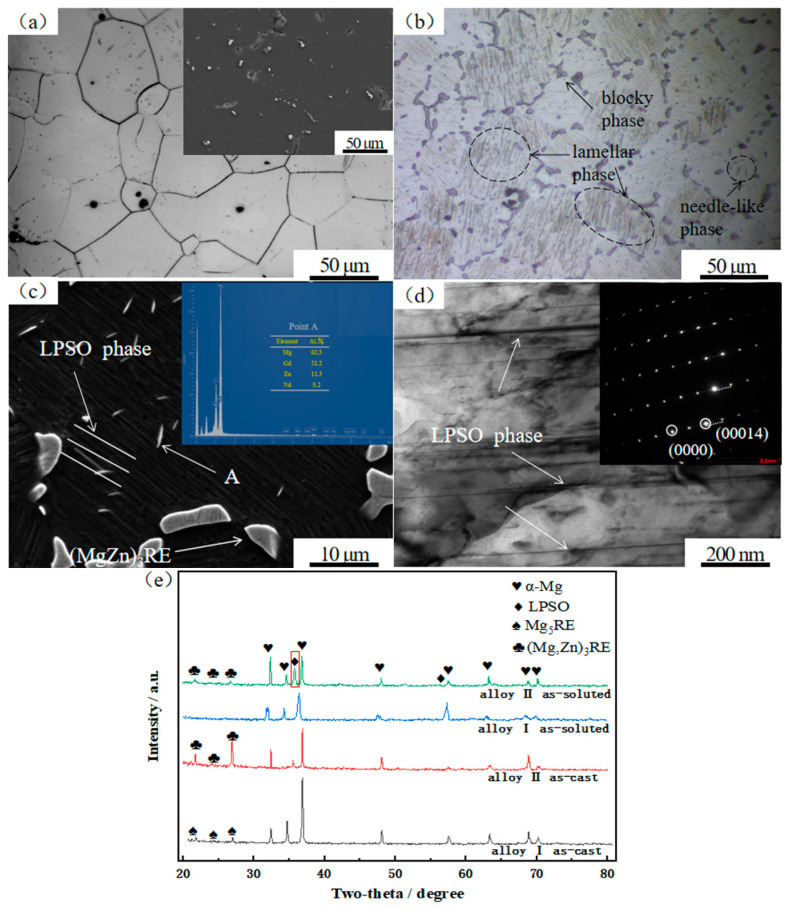
Microstructure and XRD pattern of solid solution alloys: (**a**) OM and SEM patterns of alloy I; (**b**) OM diagram of alloy II; (**c**) SEM and EDS results for alloy II; (**d**) TEM and diffraction spot patterns of LPSO phase; (**e**) XRD patterns of the alloys in different states.

Figure 3a,b, respectively, show the metallographic microstructures of alloy I and alloy II after solid solution formation. It can be seen in Figure 3a that the secondary phase (Mg_5_RE) at the crystal boundary of the as-cast alloy I was almost completely dissolved. It can be seen in Figure 3b that the semi-continuous network-like secondary phase ((Mg,Zn)_3_RE) at the crystal boundary of the as-cast alloy II was not completely dissolved but transformed into discontinuous small blocks. At the same time, a large amount of a lamellar phase and a small amount of a needle-like phase precipitated in the crystals. According to the EDS results in Figure 3c, the small needle-like phase was still (Mg,Zn)_3_RE. According to the transmission electron microscopy (TEM) and diffraction spot patterns in Figure 3d, the parallel black lamellar phase had 13 equally spaced superstructure diffraction spots between (001)α and (002)α [16], which belonged to the typical 14H-LPSO structure. Figure 3e shows the X-ray Diffraction (XRD) patterns of the two alloys. It can be seen that the as-cast alloy I only exhibited the diffraction peaks of the α-Mg matrix and the Mg_5_RE(Gd,Nd) phase, while the as-cast alloy II showed the appearance of prominent (Mg,Zn)_3_RE(Gd,Nd) diffraction peaks. After solid solution treatment, alloy I only showed the diffraction peaks of the α-Mg matrix, while alloy II exhibited not only the diffraction peaks of the α-Mg matrix and the (Mg,Zn)_3_RE phase, but also prominent diffraction peaks of the LPSO phase, as indicated by the matrix frame of the XRD pattern. The results of the XRD analysis are consistent with those of optical microscopy (OM), SEM, and TEM.

### 3.3. Microstructure of the Aged Alloys

**Figure 4:**
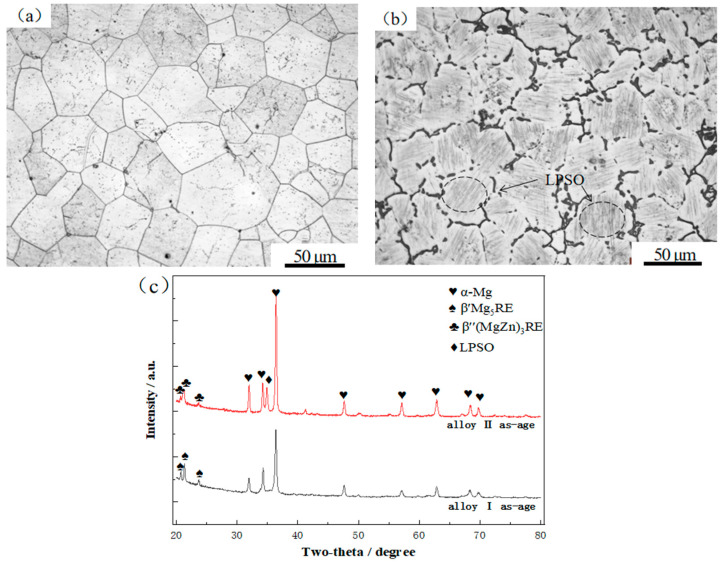
Microstructure and XRD patterns of the aged alloys: (**a**) OM diagram of alloy I; (**b**) OM diagram of alloy II; (**c**) XRD patterns of alloy I and alloy II.

Figure 4 shows the microstructure and XRD patterns of alloys I and II after the aging treatment. It can be seen in Figure 4a,c that after the aging treatment, alloy I exhibited the precipitation of a granular Mg_5_RE(Gd,Nd) phase in the crystals, which was prone to age strengthening. Comparing Figure 3b and Figure 4b, it can be seen that the amount of LPSO phase in the aged alloy II was significantly increased compared to that in the solid solution alloy II. In Figure 4b,c, it can be seen that in alloy II, a small amount of granular phase precipitated. However, due to the consumption of a certain amount of rare-earth elements during the formation of the secondary phase and the LPSO phase in alloy II, the number of precipitated phases after the aging treatment was relatively small, which could lead to a weakening of the strengthening effect.

### 3.4. Effects of the LPSO Phase on the Tensile Properties of the Mg-9Gd-2Nd-(1.5Zn)-0.5Zr Alloy at High Temperature

**Figure 5:**
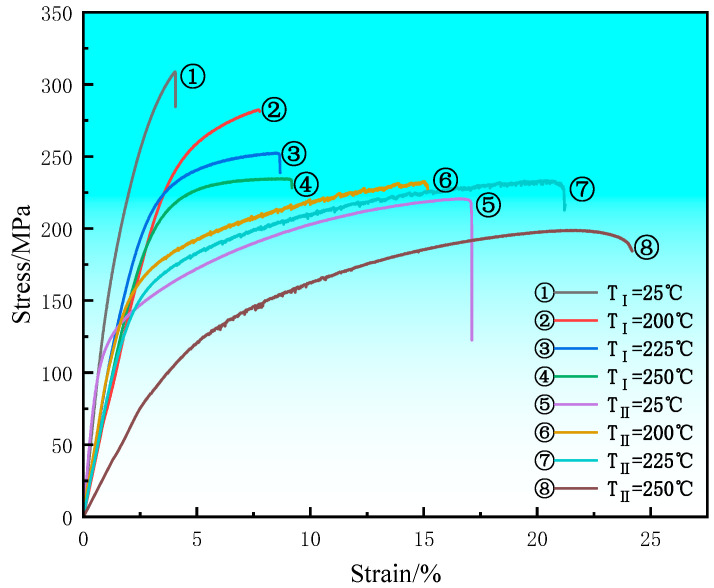
The stress–strain curves of the aged alloys at different temperatures.

Figure 5 and Table 1 show the stress–strain curves and performance data of alloys I and II at different temperatures (25 °C, 200 °C, 225 °C, and 250 °C) after the aging treatment. During the high-temperature tensile process, as the temperature gradually increased, alloy I exhibited a significant decrease in yield and tensile strength, while the elongation increased. At 250 °C, the yield and tensile strength of alloy I, respectively, decreased to 187.2 MPa and 234.2 MPa, while the elongation increased to 9.1%. The main reason for the drop in the alloy strength is that with the increase in temperature, the atomic reactivity in the crystals was enhanced, making atomic diffusion migration more likely to occur. This resulted in an increase in the vacancy density, leading to a decrease in strength. The main reason for the increase in the alloy elongation is that the structure softened at high temperatures [17], the stress concentration weakened, and the generation of microcracks was reduced. The higher the temperature, the weaker the stress concentration. At the same time, the non-basal slip of the magnesium alloy started at high temperatures [18], increasing the alloy’s ability to deform and leading to an increase in elongation.

From the tensile data in Table 1, it is clear that the strength of alloy II was lower than that of alloy I at room temperature. The main reason is that the amount of precipitated phase relatively decreased after the aging treatment, weakening the strengthening effect and resulting in a decrease in the strength of alloy II. Unlike what observed for alloy I, the yield strength of alloy II increased with the increase in the temperature between 25 °C and 200 °C. But when the temperature rose to 250 °C, its strength still experienced a significant decrease. Similar to what observed for alloy I, the elongation continued to increase along with the rise in temperature. At this time, the yield strength, tensile strength, and elongation of alloy II were 122.1 MPa, 198.8 MPa, and 24.2%, respectively. This indicates that the thermal stability of alloy II was obviously enhanced, and its plasticity was also greatly improved by the presence of the LPSO phase.

For the thermal stability of alloy II, the LPSO phase, as a hard phase, could effectively coordinate the dislocation motion [19]. Specifically, there was a semi-coherent or coherent interface between the LPSO phase and the Mg matrix. When dislocations reached the LPSO/α-Mg interface, some dislocations were suppressed and accumulated at the interface, while others entered the LPSO phase. At the LPSO/α-Mg interface, the accumulation of dislocations led to stress concentration. However, the dislocations entering the LPSO stage could continue to move, thereby enabling the LPSO phase to play a more effective role in coordinating stress and enhancing the thermodynamic stability of the alloy.

### 3.5. Mechanism of Enhancing Plasticity by the LPSO Phase

**Figure 6:**
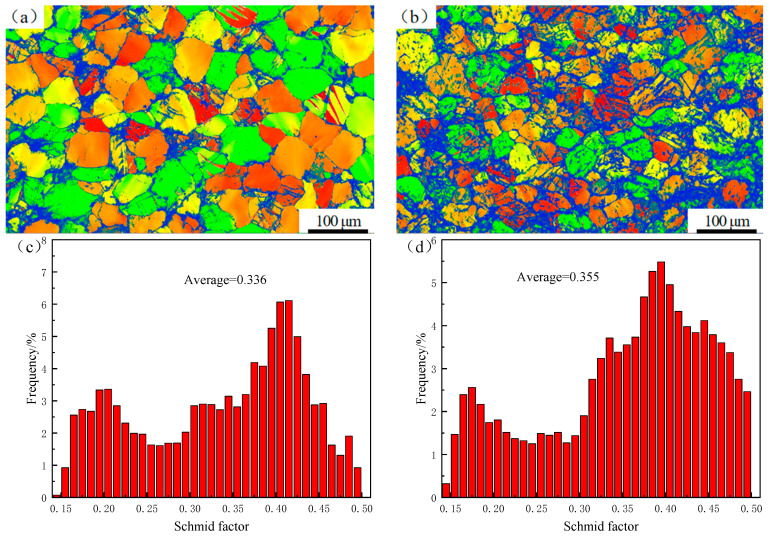
Schmid factor diagram and distribution of pyramid <c+a> Schmid factor: alloy I (**a**,**c**); alloy II (**b**,**d**).

Figure 6 shows the Schmid factor diagram and the distribution of the pyramid <c+a> Schmid factor near the fracture of alloy I and alloy II after stretching. By comparing Figure 6a,b, it can be seen that alloy II had a lower resolution due to the presence of a residual secondary phase and the LPSO phase. Figure 6c,d show the distribution of the pyramid <c+a> Schmid factor for the two alloys. It can be seen that double peaks are present in Figure 6c,d. This is due to the possible presence of tiny cracks in the specimens after stretching. Plastic deformation occurred in the region close to the cracks, resulting in a change in the orientation of the surrounding grains. However, the grain orientation far away from the cracks did not change. This situation led to the appearance of a double peak [20]. The average Schmid factor of the pyramidal <c+a> slip of alloy I and alloy II was significantly higher. This is mainly because dislocation slips are affected by the temperature. Under high-temperature conditions, dislocations are more likely to overcome the influence of precipitates and other obstacles, making it easier for the slip system to initiate the slip. At the same time, the proportion of alloy II in the high-Schmid-factor region was relatively large, leading to a higher average pyramidal <c+a> slip Schmid factor than for alloy I. This made alloy II more prone to activate pyramidal <c+a> slip under high-temperature tension. Research has found [21] that alloys with an LPSO phase are prone to activate pyramidal <c+a> slip under high-temperature conditions. Therefore, it can be inferred that alloy II underwent pyramidal <c+a> slip during high-temperature stretching, which caused dislocation diffusion, improving the material’s plastic deformation ability.

**Figure 7:**
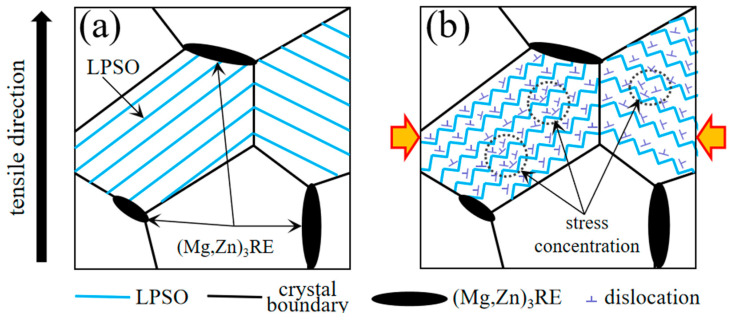
Schematic illustration of alloy plasticity influenced by the LPSO phase during tension: (**a**) microstructure of aged alloy; (**b**) layered LPSO phases that have undergone twisting and bending.

The influence mechanism of the LPSO phase on alloy plasticity is shown in Figure 7. During the stretching experiment Figure 7a, the matrix underwent deformation, as shown in Figure 7b. Pressure was generated perpendicular to the stretching direction, causing the LPSO phase to form kinks inside the crystals. In the process of kinking, the stress located near the LPSO phase caused temporary dispersion, reducing the stress it experienced and enhancing its plasticity. The critical shear stress can be expressed as the product of the Schmid coefficient and the external stress, as follows:

(1) $$\tau_{c}=m\cdot \sigma$$

In the equation, $\tau_{c}$ is the critical shear stress, m is the Schmid coefficient, and σ is the applied external stress. When the Schmid factor is high, less external stress is needed to reach the critical shear stress and initiate the slip. When a large number of dislocations move to a certain part of the LPSO phase after deformation, local stress concentration is prone to occur, causing the stress to reach the critical shearing stress (CRSS) for pyramidal slip. When the stress at the kink of the LPSO phase exceeds the critical shearing stress (CRSS), the non-basal <c+a> slip will be activated [21,22]. Therefore, the ductility and plastic deformation ability of the alloy are improved through the kinking process of the LPSO phase and the generation of the non-basal <c+a> slip.

### 3.6. Analysis of Microstructure and Morphology of the Alloy Side near a High-Temperature Tensile Fracture

**Figure 8:**
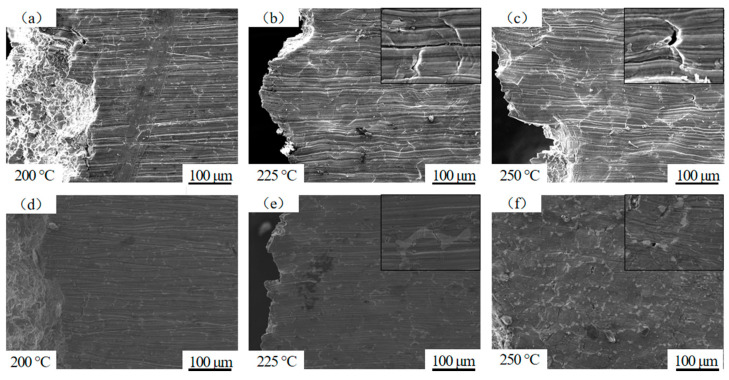
High-temperature profile morphology of alloys I and II. Alloy I: (**a**–**c**). Alloy II: (**d**–**f**).

Figure 8 shows the macroscopic microstructure morphology of the alloy side near a high-temperature tensile fracture for alloy I and alloy II at 200 °C, 225 °C, and 250 °C. By comparing the macroscopic microstructure morphology of alloy I at different temperatures in Figure 8a–c, it can be seen that with the increase in the temperature, the microstructure began to soften, the wear marks on the side of the sample were gradually bent, and the folds increased significantly. At high temperatures, the matrix structure of the alloy easily softened, which caused the grains to elongate along the stretching direction and then produce cracks [23]. During tensile testing, materials tend to develop cracks at the grain boundaries rather than inside the grains due to a decrease in the grain boundary strength. These cracks usually propagate along the grain boundaries and are perpendicular to the stretching direction. As the temperature increases, the tensile cracks increase. This is consistent with the above tensile curve showing that the higher the temperature, the lower the strength.

Figure 8d–f show the macroscopic microstructure near the fracture surface of alloy II at different temperatures. A comparison shows that there was not much difference in the microstructure of the tensile side at 200 °C and 225 °C, with a low degree of softening, no significant bending of the wear marks, and relatively fewer wrinkles. According to research [24], the elastic modulus of the LPSO phase is higher than that of the α-Mg matrix, which causes the LPSO phase to maintain elasticity even when the α-Mg matrix undergoes deformation, thereby hindering deformation. When the temperature reached 250 °C, the grains underwent significant deformation, wrinkles began to appear in large numbers, and the secondary phase began to soften, causing cracks to begin to form at the crystal boundaries. The softening of the microstructure led to a weakening of the stress concentration, further increasing the occurrence of cracks and reducing the strength. The presence of the LPSO phase within the crystals led to pyramidal slip, which improved the plasticity of the alloy, consistent with the observed tensile curve characteristics.

## 4. Conclusions

1. The cast alloy I mainly consisted of an α-Mg matrix and a Mg_5_RE phase. After adding Zn, the Mg_5_RE phase in the cast alloy transformed into a (Mg,Zn)_3_RE phase, and the morphology changed from an irregular strip to a semi-continuous network.
2. After solid solution treatment, the secondary phase at the crystal boundaries of alloy I was almost completely dissolved in the matrix, while there were still residual secondary phases at the crystal boundaries of alloy II, and layer-like LPSO phases were generated under the interaction of Zn and the rare-earth elements.
3. After the aging treatment, alloy I formed precipitates within the crystals, resulting in aging strengthening, and alloy II, due to the consumption of the RE elements by the LPSO phase, inevitably formed fewer precipitates, which weakened the strengthening effect.
4. During the high-temperature tensile test, as the temperature gradually increased, the yield and tensile strength of alloy I decreased by 27 MPa and 74 MPa, respectively, and the elongation gradually increased to 9.1%. However, the decreasing trend for the tensile strength of alloy II significantly weakened, and the tensile strength decreased by 45 MPa. At the same time, the elongation was also higher than that of alloy I, increasing to 24.2%. This is mainly attributed to the ability of the LPSO phase to coordinate the stress and the activation of the pyramidal <c+a> slip under high-temperature conditions.
5. In the high-temperature tensile structure, the side structure of alloy I softened, wrinkles increased, and there were microcracks perpendicular to the tensile direction, which became larger and more numerous with the increasing temperature. However, the softening degree of alloy II was not significant, and there were no significant wrinkles. Partial wrinkles and cracks only appeared when the temperature reached 250 °C.

In conclusion, alloy II has the potential for widespread applications in the field of high-plasticity magnesium alloys. However, due to its limited strength, future research could focus on enhancing its strength while maintaining the presence of the lamellar LPSO phase in the alloy.

## Figures and Tables

**Table 1:** The tensile property data of the aged alloys at different temperatures.

Temperature (°C)		YS (MPa)	UTS (MPa)	EL (%)
Mg-9Gd-2Nd- 0.5Zr(I)	25	215.1 ± 5.1	308.7 ± 3.2	2.7 ± 0.1
	200	213.7 ± 4.2	281.9 ± 2.5	7.8 ± 0.2
	225	203.1 ± 4.9	251.1 ± 2.7	8.6 ± 0.2
	250	187.2 ± 3.7	234.2 ± 2.4	9.1 ± 0.3
Mg-9Gd-2Nd- 1.5Zn-0.5Zr(II)	25	150.7 ± 5.6	243.3 ± 3.1	11.8 ± 0.4
	200	153.7 ± 4.2	232.7 ± 2.9	15.1 ± 0.4
	225	146.1 ± 5.6	233.2 ± 3.3	20.1 ± 0.6
	250	122.1 ± 5.1	198.9 ± 3.0	24.2 ± 0.7

## Data Availability

The authors declare that no new data were created.

## References

[1] Chen T., Chen Z.Y., Shao J.B., Wang R.K., Mao L.H., Liu C.M. (2019). The role of long-period stacking ordered phases in the deformation behavior of a strong textured Mg-Zn-Gd-Y-Zr alloy sheet processed by hot extrusion. Mater. Sci. Eng. A.

[2] Jin X.Z., Xu W.C., Li K.L., Zeng X.Q., Shan D.B. (2018). Influence of heat treatment on the evolution of microstructure and mechanical properties of Mg-7Gd-5Y-0.6Zn-0.8Zr magnesium alloy. Mater. Sci. Eng. A.

[3] Peng Q.M., Wu Y.M., Fang D.Q., Meng J., Wang L.M. (2007). Microstructures and mechanical properties of Mg-8Gd-0.6Zr-xNd (x = 0, 1, 2 and 3 mass%) alloys. J. Mater. Sci..

[4] Negishi Y., Iwasawa S., Kamado S., Kojima Y., Ninomiya R. (1994). Effect of yttrium and neodymium additions on aging characteristics and high temperature tensile properties of Mg-10 mass%Gd and Mg-10 mass%Dy alloys. J. Jpn. Inst. Light Met..

[5] Zheng K.Y., Dong J., Zeng X.Q., Ding W.J. (2007). Precipitation and its effect on the mechanical properties of a cast Mg–Gd–Nd–Zr alloy. Mater. Sci. Eng. A.

[6] Yang X.D., Zhou X.J., Yu S.L., Zhang J., Lu X.Z., Chen X.M., Lu L.W., Huang W.Y., Liu Y.R. (2022). Tensile behavior at various temperatures of the Mg-Gd-Y-Zn-Zr alloys with different initial morphologies of LPSO phases prior to extrusion. Mater. Sci. Eng. A.

[7] Chuang W.S., Hsieh C.H., Huang J.C., Lin P.H., Takagi K., Mine Y., Takashima K. (2017). Relation between sample size and deformation mechanism in Mg-Zn-Y 18R-LPSO single crystals. Intermetallics.

[8] Hagihara K., Kinoshita A., Sugino Y., Yamasaki M., Kawamura Y., Yasuda H.Y., Umakoshi Y. (2010). Plastic deformation behavior of Mg89Zn4Y7 extruded alloy composed of long-period stacking ordered phase. Intermetallics.

[9] Zhou X.J., Liu C.M., Gao Y.H., Jiang S.N., Liu W.H., Lu L.W. (2018). Microstructure and mechanical properties of extruded Mg-Gd-Y-Zn-Zr alloys filled with intragranular LPSO phases. Mater. Charact..

[10] Liu H., Huang H., Wang C., Sun J.P., Bai J., Xue F., Ma A.B., Chen X.B. (2019). Recent Advances in LPSO-Containing Wrought Magnesium Alloys: Relationships Between Processing, Microstructure, and Mechanical Properties. JOM.

[11] Zhao T.S., Hu Y.B., Pan F.S., He B., Guan M.S., Yuan Y., Tang A.T. (2019). Effect of Zn Content on the Microstructure and Mechanical Properties of Mg–Al–Sn–Mn Alloys. Materials.

[12] Ding W.J., Wu Y.J., Peng L.M., Zeng X.Q., Yuan G.Y., Lin D.L. (2009). Formation of 14H-type long period stacking ordered structure in the as-cast and solid solution treated Mg-Gd-Zn-Zr alloys. J. Mater. Res..

[13] Wu Y.J., Lin D.L., Zeng X.Q., Peng L.M., Ding W.J. (2009). Formation of a lamellar 14H-type long period stacking ordered structure in an as-cast Mg-Gd-Zn-Zr alloy. J. Mater. Sci..

[14] Zhao S.S., Xu Y.J., Geng C.G., Lin X.P., Tang Q., Dong Y. (2022). High temperature mechanical properties and strain hardening mechanism of directionally solidified Mg-Gd-Y alloy. Mater. Sci. Eng. A.

[15] Cheng R.T., Yan L.P., Li X.K., Feng Z., Su G. (2024). Research on microstructure and mechanical properties of hot extruded Mg-Gd-Y-Sm-Zr alloy at room and high temperatures. J. Mater. Res. Technol..

[16] Robson J.D., Henry D.T., Davis B. (2009). Particle effects on recrystallization in magnesium-manganese alloys: Particle-stimulated nucleation. Acta Mater..

[17] Zhu S.M., Easton M.A., Abbott T.B., Nie J.F., Dargusch M.S., Hort N., Gibson M.A. (2015). Evaluation of Magnesium Die-Casting Alloys for Elevated Temperature Applications: Microstructure, Tensile Properties, and Creep Resistance. Metall. Mater. Trans..

[18] Yan L.P., Li Q.A., Zhu L.M., Chen X.Y., Yang L.D., Chen J., Li W.H. (2021). Investigation of hot extruded GW84 alloy on high temperature tensile properties and microstructure evolution. J. Mater. Res. Technol..

[19] Dang C., Wang J.F., Wang J.X., Yu D., Zheng W.X., Xu C.B., Wang Z.H., Feng L., Chen X.H., Pan F.S. (2024). Preparation of high-strength and high-damping Mg-Gd-Y-Zn-Zr-Nd alloy by regulating LPSO and β′ phases. Trans. Nonferrous Met. Soc. China.

[20] Tang J.H., Zhang Y.D., Ye L.Y., Qu M., Wu J.S., Zhang Z., Liu S.D., Deng Y.L. (2019). Effect of Grain Boundary and Crystallographic Orientation on the Stress Corrosion Behavior of an Al-Zn-Mg Alloy. J. Mater. Eng. Perform..

[21] Kim J.K., Sandlöbes S., Raabe D. (2015). On the room temperature deformation mechanisms of a Mg–Y–Zn alloy with long-period-stacking-ordered structures. Acta Mater..

[22] Luan S.Y., Zhang L., Chen L.J., Li W., Wang J.H., Jin P.P. (2023). The influence of the LPSO on the deformation mechanisms and tensile properties at elevated temperatures of the Mg-Gd-Zn-Mn alloys. J. Mater. Res. Technol..

[23] Pettersen G., Westengen H., HøIer R., Lohne O. (1996). Microstructure of a pressure die cast magnesium—4wt.% aluminium alloy modified with rare earth additions. Mater. Sci. Eng. A.

[24] Lv B.J., Peng J., Peng Y., Tang A.T., Pan F.S. (2013). The effect of LPSO phase on hot deformation behavior and dynamic recrystallization evolution of Mg–2.0Zn–0.3Zr–5.8Y alloy. Mater. Sci. Eng. A.

